# A global electricity transmission database for energy system modelling

**DOI:** 10.1016/j.dib.2024.110420

**Published:** 2024-04-15

**Authors:** Maarten Brinkerink, Gordon Sherman, Simone Osei-Owusu, Reema Mohanty, Aman Majid, Trevor Barnes, Taco Niet, Abhishek Shivakumar, Erin Mayfield

**Affiliations:** a Dartmouth College, Thayer School of Engineering, Hanover, NH, USA; b University College London, Bartlett School of Environment, Energy & Resources, London, UK; c Climate Compatible Growth, London, UK; d TransitionZero, London, UK; e Oxford Programme for Sustainable Infrastructure Systems (OPSIS), University of Oxford, Oxford, UK; f School of Sustainable Energy Engineering, Simon Fraser University, Surrey, BC, Canada

**Keywords:** Interconnectors, Transmission, Electricity grid, Power systems, Energy system modelling, Global, Energy policy

## Abstract

Energy system modelling can be used to provide scenario-based insights in energy system transition pathways. However, data accessibility is a common barrier for the model representation of energy systems, both regarding existing infrastructure, as well as planned developments consistent with current policies. This paper describes the ‘Global Transmission Database’, the first global dataset covering existing and planned electricity transmission developments between countries and selected regions. The dataset can be used as a starting point for the representation of cross-regional electricity grids globally in energy system models and other computational tools. All data is collected from publicly available sources and combined into a single machine-readable format for convenient application.

Specifications Table

**Table:** 

Subject	Energy
Specific subject area	Energy System Modelling
Data format	Raw, Analyzed, Filtered
Type of data	Table, Figure
Data collection	Where available, data is collected from annual reports, policy reports or websites from electricity system operators or other regulatory entities. Alternatively, if data can not be found from these sources, data is collected from academic articles and modelling databases, or referenced newspaper articles and webpages. All retrieved data is from open and accessible data sources. Raw data is mostly used, with some modifications (e.g. conversion of miles into kilometres) to ensure consistency. Inclusion and exclusion of data entries is based on author judgement with supporting information provided.
Data source location	A comprehensive overview of used sources for raw data entries is provided in the data repository (Zenodo).
Data accessibility	Repository name: Zenodo Data identification number: 10.5281/zenodo.10063445 Direct URL to data: https://zenodo.org/doi/10.5281/zenodo.10063445

## 1 Value of the Data

• Provides analysts with an entry-level global dataset of existing and planned transboundary electricity transmission capacities.
• Formatted to be used in energy system models or other computational tools as a starting point for the representation of cross-regional electricity grids globally.
• Collates data from a range of sources in multiple languages and harmonises data in a single machine-readable format, thereby removing data accessibility barriers.

## 2 Background

The use of electricity transmission infrastructure to support decarbonization efforts is becoming more prominent. In the International Energy Agency's Net Zero Roadmap [1], global investment needs for transmission and distribution grids are projected to double by 2030 to USD 680 billion annually and to more than 1 trillion annually by 2040. In addition, there has been significant growth in the number and size of interconnections between regions and countries [2]. There are several computational energy and electric power system tools capable of evaluating interconnected electricity systems around the globe (e.g. [3], [4], [5]). For these computational tools, it is essential that reliable and comprehensive data is used to represent existing systems as well as to include planned developments that are consistent with current policies. Detailed global open datasets exist for other power system components such as for power plants [6,7] and electricity storage assets [8], yet current open data for electricity transmission infrastructure tends to be limited to a subset of countries or regions (e.g. [9]) or are usually not provided in standardised machine-readable formats (e.g. [10]). Global datasets that do exist, such as the OpenStreetMap project [11], provide data solely in terms of voltage (kV) on a line-by-line basis. However, energy system models often do not model individual transmission lines, but rather require more aggregated data in terms of total transmission capacity (MW) between countries or regions. These existing global datasets therefore require simplified assumptions to convert data into a usable format for energy system models. Furthermore, these datasets tend to only include existing infrastructure, neglecting planned infrastructure projects.

## 3 Data Description

**Fig. 1:**
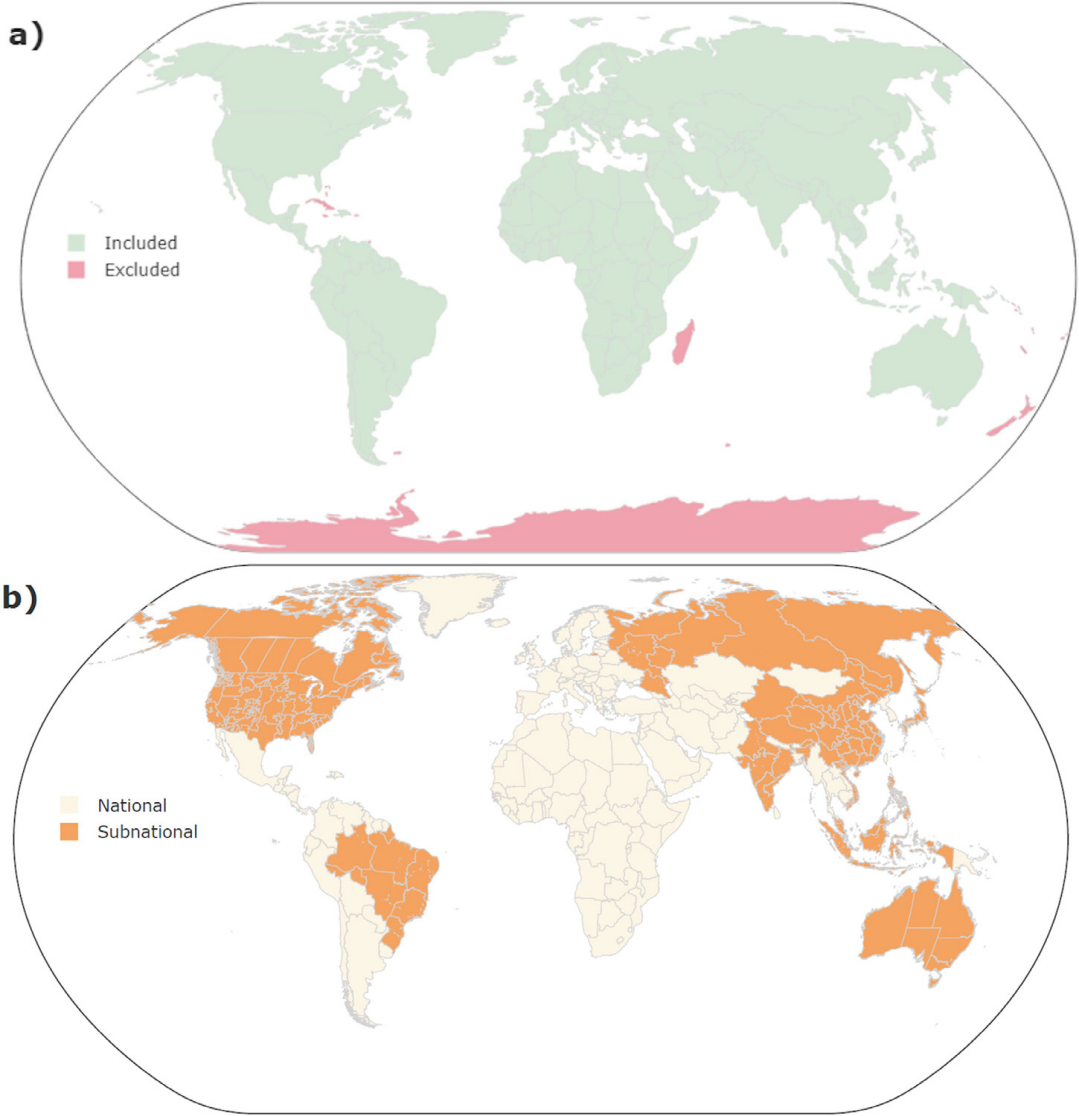
Map showing (a) the countries that are included and excluded within the dataset and (b) the countries that have sub-national scale representation (dark orange) including their regional division.

This Data in Brief describes the ‘Global Transmission Database’, the first global dataset covering existing and planned electricity transmission capacities between countries and selected regions. As highlighted in Fig. 1a, all transmission pathways between land-connected countries are included, as well as island countries that either have existing cross-border interconnections (e.g. Malta with Italy), have planned interconnections (e.g. Sri Lanka with India), or have been discussed in the past to be interconnected to other countries (e.g. Iceland and Great Britain). Most countries are represented as a single region, with a few larger countries represented by individual admin-1^1^ regions (Australia, Canada^2^, China), aggregation of admin-1 regions (Brazil, India, Indonesia, Japan, Malaysia, Philippines, Russia, Vietnam), or by system operator (United States^3^) depending on data availability. The spatial representation of the Global Transmission Database is shown in Fig. 1b. Note that all Figures can be recreated and viewed in high resolution within the relevant GitHub repository supporting this Data in Brief [12].

The dataset consists of four Comma-Separated Values (CSV) files that cover the global transmission data in terms of aggregated capacities per transmission pathway (MW), and where available line distances (km) and voltages (kV). This includes two files representing existing and planned transmission capacities with regional representation where applicable (‘GTD-v1.0_regional_existing.csv’ and ‘GTD-v1.0_regional_planned.csv’) and two files with existing and planned capacities aggregated to the country-level for user convenience (‘GTD-v1.0_national_existing.csv’ and ‘GTD-v1.0_national_planned.csv’). Also included is a mapper file that provides a textual description of the spatial representation of the transmission data (‘GTD_mapping.csv’). Table 1 includes column descriptions of the four transmission data CSV's, and Table 2 includes column descriptions of the mapper file.

**Table 1:** Column description of the capacity files in the dataset repository.

Column	Description
from_country to_country	The three letter alpha-3 country code. For example, any cross-border transmission data related to Afghanistan will be represented by ‘AFG’. The ‘from_country’ and ‘to_country’ columns are alphabetically determined and are merely used as identifiers. This does not reflect that electricity can only flow from direction ‘from’ to direction ‘to’. The countries and regions are split into ‘from’ and ‘to’ given that the identified maximum directional flows on specific transmission lines and pathways can be heterogeneous.
from_region to_region	The name abbreviation for a specific sub-country region for which transmission data is represented. This is a five letter code that consists of I) the three letter country code and II) a two letter sub-country specific code OR the letters ‘XX’ indicating that for the specific country no regional subdivision is included. For example, any cross-border transmission data related to Afghanistan (AFG) will be represented by ‘AFGXX’ whereas cross-regional data for the British Columbia (BC) province in Canada (CAN) will be represented by ‘CANBC’.
pathway	The name for a specific cross-border or cross-regional transmission pathway. This is a nine letter code that consists of I) the letters ‘TRN’ (transmission), II) the region_from and III) the region_to abbreviations. For example, cross-border transmission data between Afghanistan (‘AFGXX’) and Iran (‘IRNXX’) is represented as ‘TRNAFGXXIRNXX’ and cross-regional transmission data between Alberta (AB) and British Columbia (BC) as ‘TRNCANABCANBC’.
max_flow	The maximum unidirectional electricity transmission capacity (MW) in the direction ‘from_region’ to ‘to_region’. The provided value is given as total capacity. This can be based on the combined capacity of one-to-many individual transmission lines between the two respective countries or sub-country regions. The provided existing capacities reflect capacities as of 2023 or the latest Zenodo [14] release in case of potential future dataset updates. Planned capacities are separated by expected commissioning year (‘year_planned’ column) by means of individual rows.
max_counter_flow	The maximum unidirectional electricity transmission capacity (MW) in the reversed direction ‘to_region’ to ‘from_region’. As stated earlier, capacities are separated by direction given that the identified maximum directional flows on specific transmission lines and pathways can be heterogeneous.
voltage	Where available, data regarding the voltage (kV) of existing or planned transmission lines. The provided values are by individual transmission line with “;” as delimiter and “/” as indicator for split voltages on different parts of the line. E.g. the ‘TRNARGXXPRYXX’ pathway has ‘220;500;220/132’ as entry, which implies that data exists for three lines, one at 220 kV, one at 500 kV and one with split voltages of 220 kV and 132 kV on different parts of the line. Note that the provided voltage data is not necessarily complete, additional lines can exist for the specific pathway yet respective values have not been found in the assessed sources.
distance	Where available, data regarding the distance (km) of existing or planned transmission lines. Provided data corresponds to the data as given in the ‘voltage’ column. For example, the ‘TRNARGXXPRYXX’ pathway has ‘;;35’ as an entry which implies that only data for the third entry (‘220/123’) in the ‘voltage’ column has been retrieved.
year_planned	Only relevant for the planned capacities, this column represents the expected commissioning year for the provided transmission capacity. The column is left empty if no specific commissioning year has been found.
source_1 source_2	Relevant sources as used for the derived values. Note that the sources as used for the existing capacities can be different compared to the sources as used for the planned capacities.
assumptions	Where applicable, this column includes any relevant assumptions as made to derive the respective dataset entry. In most instances, values are included from secondary sources without manipulation, however additional assumptions are often required. For example, to include a singular value when potential capacities are provided in a range, or to argue why specific projects are included or excluded.
other_notes	Any relevant information related to the included data that does not correspond to assumptions made by the authors.

**Table 2:** Column description of the ‘GTD_mapping.csv’ file in the dataset repository.

Column	Description
country	The three letter alpha-3 country code.
region	The name abbreviation for a specific sub-country region for which transmission data is represented. This is a five letter code that consists of I) the three letter country code and II) a two letter sub-country specific code OR the letters ‘XX’ indicating that for the specific country no regional subdivision is included. For example, any cross-border transmission data related to Afghanistan (AFG) will be represented by ‘AFGXX’ whereas cross-regional data for the British Columbia (BC) province in Canada (CAN) will be represented by ‘CANBC’.
region_name	Relevant for countries with regional representation, this column indicates the full regional name for the specific entry. Names can correspond to individual admin-1 regions (e.g. British Columbia) or can have custom names to e.g. reflect a specific geographical area of a country (e.g. Brazil Center-North).
region_admin-1	Relevant for regions that are based on a single, or groups of, admin-1 regions, this column maps all admin-1 regions that are part of the specific entry with “;” as delimiter.
region_custom	Relevant for regions with custom spatial representation, this column describes the custom region entry. For example, for the United States, it provides the name of the relevant balancing authority.

**Fig. 2:**
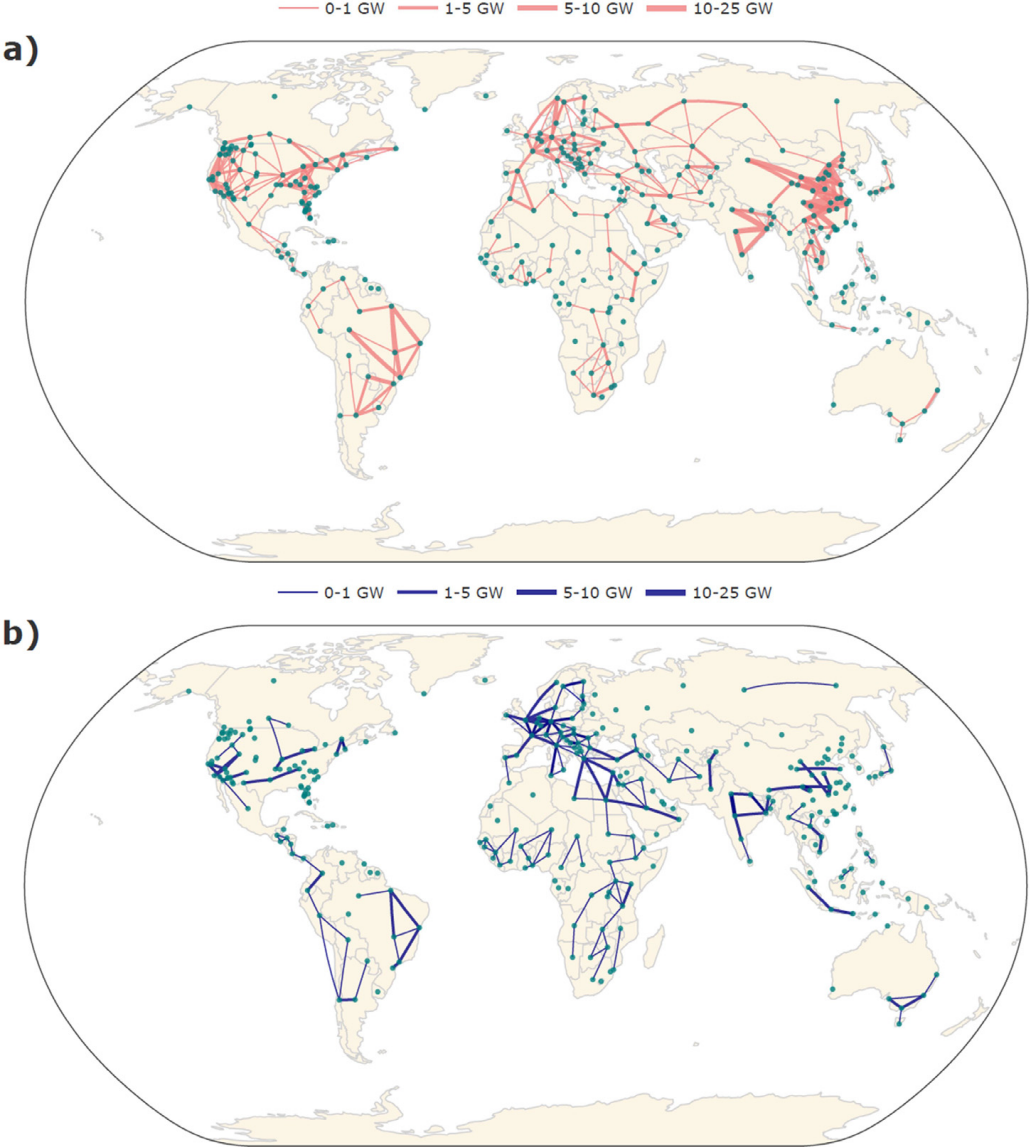
Map showing (a) the existing transmission line capacity and (b) the total planned transmission line capacity by region based on the dataset. High resolution and interactive versions of the transmission maps can be recreated and found on the relevant GitHub repository supporting this Data in Brief [12].

**Fig. 3:**
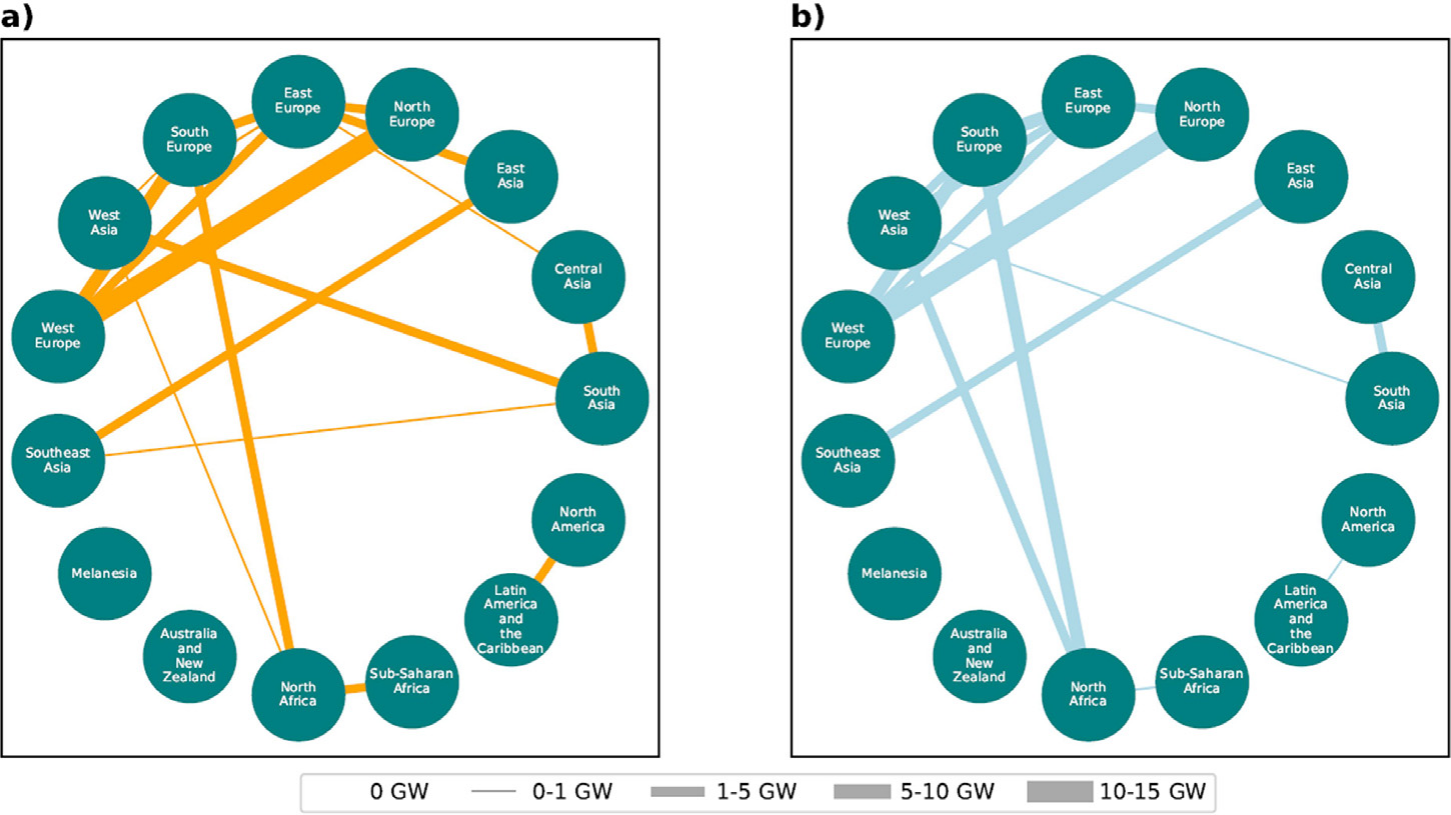
Existing (a) and planned (b) transmission capacity (GW) between world subregions.

Fig. 2a, b depict the existing and planned transmission capacities as included in the dataset. Note that the location of the start and end points of the different transmission pathways is meant to be indicative and does not reflect actual transmission distances or routes. Overall, the largest existing capacities between countries are located within Europe, South America, and between Canada and the United States. At a regional level, large transmission capacities are prominent within China, India and Brazil. Generally, countries and regions within these areas also have the largest expected planned transmission capacities. At a smaller scale, though not for the countries involved, substantial build outs of transmission infrastructure are planned in Sub-Saharan Africa, Latin America, the Middle East and South-East Asia. Fig. 3 shows that significant transmission developments between different regions in Europe are planned, as well as additional transmission integration with North Africa.

The provided data can be used as a starting point for the representation of cross-regional electricity grids globally in energy system models or other planning tools. As an example, the dataset has been used for a modelling study assessing potential strategies for the global electricity system to reach net-zero by 2040 with a focus on determining the cost-benefits of global transmission integration [13].

## 4 Experimental Design, Materials and Methods

The main applied methods to create the dataset are a literature review regarding available sources for transmission data and manual data scraping to port the data into a standardised and machine-readable format. We prioritised data sourced from utilities (e.g. [10]) or relevant governmental agencies (e.g. [15]), but where data could not be retrieved from these entities, we sourced values from other organisational reports (e.g. [16]), academic research (e.g. [17]), or news reports (e.g. [18]). Sources can range from datasets that cover multiple countries (e.g. [9]) to a specific transmission project (e.g. [16]). The majority of data collected were in units of MW transmission capacity and did not require additional data transformations. However, in certain cases, additional assumptions were required to harmonise data. For example, this included assumptions regarding how to represent capacities that were reported as ranges, whether to include or exclude specific projects, or whether reported capacities are bilateral or unilateral to e.g. facilitate the cross-border supply from a specific power plant.

For a subset of data entries, namely for transmission capacities between balancing authorities in the United States, as well as for capacities between a number of Eastern European countries, we use historical hourly electricity trade values (2019-2023) as a proxy to determine maximum unilateral transmission capacities. The reporting of transmission data in the United States is relatively scarce due to national security reasons [19]. Rather than attempting to retrieve the data by going into individual reports per balancing authority (63 in total within the dataset), we opt to use an alternate approach by making use of electricity trade data from the United States Energy Information Administration (EIA) [20]. This allows us to create standardised and replicable dataset entries. The supporting script to derive transmission capacities based on historical trade values for the United States can be found on the relevant GitHub repository supporting this Data in Brief [12]. Table 3 summarizes the main applied methods to derive the dataset entries. All relevant assumptions per individual entry are documented within the dataset itself [14].

**Table 3:** Summary table applied methods and data sources.

Main method		

The main method used to derive input data for the dataset has been manual scraping and porting the data into a standardised and machine-readable format. We prioritised data sourced from utilities or relevant governmental agencies, but where data could not be retrieved from these entities, we sourced values from other organisational reports, academic research, or news agencies. Additional assumptions were often required to harmonise the data. Detailed assumptions per dataset entry can be found within the dataset [14].		

**Continent**	**Other key methods**	**Data sources (n)**

Africa	-	90
Asia	Given data limitations for transmission within China, planned capacities only cover ultra-high voltage transmission line projects (800 kV and higher).	111
Europe	For a number of countries within Eastern Europe (Belarus, Kosovo, Moldova), some or all existing transmission capacities to neighbouring countries are determined by using historical (2021-2023) hourly electricity trade values as a proxy to determine maximum unilateral transmission capacities.	27
North America	Regarding existing capacities between balancing authorities in the United States, historical (2019-2023) hourly electricity trade values [20] are used as a proxy to determine maximum unilateral transmission capacities. Given data limitations for transmission within the United States, planned capacities only cover transmission lines with voltages higher than 230 kV.	102
Oceania	-	14
South America	-	12

## Limitations

Until now, transmission data for countries and regions globally has been fragmented and with deviating quality of source material. Although this global dataset alleviates the first issue, the difference in data quality remains a limitation. It remains up to the user's judgement to decide on the inclusion or exclusion of specific dataset entries depending on the research question. Secondly, this dataset is designed for application in energy system models and other planning tools with limited applicability for power flow studies in operational models. Thirdly, it is important to realise that the provided capacities can be different in technical terms depending on the source material. For example, capacities can be reported as nominal capacity, as net transfer capacity for a specific season or operating condition, or as allocated capacity following a trade agreement or other constraint. This means that in some instances the actual transmission capacity could be higher or lower, which cannot be confirmed based on the assessed sources. Fourth, the dataset is limited with respect to the amount of countries that have regional transmission capacities represented. Fifth, we do not distinguish between transmission planning stages due to data limitations. Finally, it may be difficult to replicate or update the dataset because of the large time and resource requirements. Artificial intelligence based approaches that could assist with automated data identification and data scraping might be useful in this context, yet these are complicated to apply due to the wide variety of data sources in different formats and languages.

## Ethics Statement

The authors have read and follow the ethical requirements for publication in Data in Brief and confirm that the current work does not involve human subjects, animal experiments, or any data collected from social media platforms.

## CRediT authorship contribution statement

**Maarten Brinkerink:** Conceptualization, Data curation, Investigation, Methodology, Software, Supervision, Validation, Writing – original draft, Writing – review & editing. **Gordon Sherman:** Data curation, Investigation. **Simone Osei-Owusu:** Conceptualization, Data curation, Methodology, Investigation, Writing – review & editing. **Reema Mohanty:** Data curation, Investigation, Writing – review & editing. **Aman Majid:** Data curation, Methodology, Investigation, Software, Visualization, Writing – review & editing. **Trevor Barnes:** Conceptualization, Data curation, Investigation, Methodology, Software, Visualization, Writing – review & editing. **Taco Niet:** Conceptualization, Supervision, Writing – review & editing. **Abhishek Shivakumar:** Conceptualization, Data curation, Investigation, Methodology, Writing – review & editing. **Erin Mayfield:** Conceptualization, Supervision, Writing – review & editing.

## Data Availability

Global Transmission Database (Original data) (Zenodo)

## Footnotes

1 The largest subnational administrative unit of a country such as states, provinces or prefectures.

2 With the exception of i) the Northwest Territories, Yukon, and Nunavut and ii) New Brunswick, Nova Scotia and Prince Edward Island, which are grouped together into a single region.

3 Continental United States, with Alaska treated as a single region.
